# The association between HIV self-test awareness and recent HIV testing uptake in the male population in Gambia: data analysis from 2019–2020 demographic and health survey

**DOI:** 10.1186/s12879-023-08254-4

**Published:** 2023-05-26

**Authors:** Phyumar Soe, Lisa G. Johnston, Jean Damascene Makuza, Mohammad Ehsanul Karim

**Affiliations:** 1 School of Population and Public Health, University of British Columbia, Vancouver, BC Canada; 2 LGJ Consultants, Inc., Valencia, Spain; 3 School of Public Health and Tropical Medicine, Tulane University, New Orleans, Loiusiana Spain; 4 Clinical Prevention Services, British Columbia Centre for Disease Control, Vancouver, BC Canada; 5 Centre for Health Evaluation and Outcome Sciences, St. Paul’s Hospital, Vancouver, Canada

**Keywords:** HIV, Demographic and health survey, HIV self-testing awareness, HIV testing uptake, Gambia

## Abstract

**Background:**

The Gambian Ministry of Health is supportive of HIV self-testing (HIVST) and HIVST initiatives are being piloted as an additional strategy to increase HIV testing for individuals not currently reached by existing services, particularly men. This study aimed to determine awareness of HIVST among Gambian men, and whether prior awareness of HIVST is associated with recent HIV testing uptake.

**Methods:**

We used men’s cross-sectional data from the 2019–2020 Gambian Demographic and Health Survey. We employed design-adjusted multivariable logistic regression to examine the association between HIVST awareness and recent HIV testing. Propensity-score weighting was conducted as sensitivity analyses.

**Results:**

Of 3,308 Gambian men included in the study, 11% (372) were aware of HIVST and 16% (450) received HIV testing in the last 12 months. In the design-adjusted multivariable analysis, men who were aware of HIVST had 1.76 times (95% confidence interval: 1.26–2.45) the odds of having an HIV test in the last 12 months, compared to those who were not aware of HIVST. Sensitivity analyses revealed similar findings.

**Conclusion:**

Awareness of HIVST may help increase the uptake of HIV testing among men in Gambia. This finding highlights HIVST awareness-raising activities to be an important intervention for nationwide HIVST program planning and implementation in Gambia.

## Introduction

In 2021, an estimated 38.4 million people were living with human immunodeficiency virus infection (HIV) worldwide, with 1.5 million new infections and 650,000 AIDS-related deaths [1]. Sub-Saharan Africa (SSA) bears two-thirds of the global HIV/AIDS burden [2]. Despite the substantial progress in combating HIV in SSA, the vast majority of countries missed the 2020 global targets set by The Joint United Nations Programme on HIV/AIDS [2]. The global targets envisioned that 90% of people living with HIV know their HIV status, 90% who know their HIV-positive status are accessing treatment and 90% on treatment have suppressed viral loads by 2020 [2]. The current global targets are even more strict with a call to achieve 95% of these targets by 2030 [3]. Low HIV testing coverage and knowledge of status, as well as suboptimal treatment are key gaps in countries’ responses to HIV, particularly among men and young people residing in SSA [4].

Gambia is one of the smallest countries in SSA in which HIV prevalence among adults aged 15 to 49 is estimated to be 1.7% (men: 1.3%; women: 2.1%), with approximately 25,000 people living with HIV/AIDS in 2021 [5]. UNAIDS suggests that men who have sex with men comprise no less than 1.2% of the adult male population [6]. In Gambia, the most recent data on men who have sex with men, reports an HIV prevalence of 35.5% [7]. Although the Gambian National AIDS Control Program has increased HIV counselling and testing services substantially, testing coverage remains low, with less than 40% of the those living with HIV knowing their status in 2020 [7]. According to the 2020 Gambia Demographic and Health Survey (GDHS), there is a gender difference between women and men (aged 15–49) in HIV testing uptake; 39% of women, compared with 25% of men, reported ever having an HIV test. This may be linked to the fact that all pregnant Gambian women attending antenatal clinics are routinely offered HIV tests [8]. However, gender disparity in HIV testing was identified in almost all SSA countries [9, 10], with several key individual barriers related to men’s low uptake of HIV testing, including a lack of HIV knowledge, social norms around masculinity, fear of having a positive HIV test and negative consequences associated with testing results, stigma, confidentiality, and loss of job opportunities [11–15]. Thus, it is important to increase HIV testing uptake among Gambian men who have never had an HIV test in order to accelerate the UNAIDS targets to end AIDS by 2030.

HIV self-testing (HIVST) is a convenient and confidential option that enables individuals to test themselves for HIV and receive results immediately [16]. In 2016, the World Health Organization (WHO) recommended HIVST as an additional reliable testing approach to reach those who may not test otherwise, and a reactive self-test result should be followed by further testing and confirmation by a trained provider [16]. Several randomized control and pragmatic trials conducted worldwide have shown a significant increase in the uptake of HIVST compared to routine HIV testing services upon providing informed choices for different testing options [16–18]. Globally, many countries are supportive of HIVST policies, and implementation is growing rapidly [19]. Studies conducted in SSA found poor knowledge of HIVST among men, and revealed men’s perspective and willingness to use HIVST, as well as promote its usage among male peers once they were aware of HIVST benefits [20–24].

The HIVST program in Gambia is in development and some initiatives are being piloted among those who are at higher risk of HIV [7]. Before implementing nationwide HIVST programs within the country, it is important to assess HIVST awareness among adult Gambian men, and whether this prior awareness is associated with recent HIV testing uptake in general. The recent 2020 GDHS offers the opportunity to assess the relationship between HIVST awareness and HIV testing uptake using a nationally representative sample. We hypothesize that men who are aware of HIVST are more likely to uptake any kind of HIV testing. The findings from this analysis will inform the country’s policy for the adoption and scale-up of HIVST as part of Gambia’s national HIV response.

## Methods

### Study design and data source

This analysis used cross-sectional data from the 2019–2020 GDHS implemented by the Gambia Bureau of Statistics. This nationally representative survey included participants aged 15 years and above residing in all eight Local Government Areas of Gambia [25]. A representative sample of households was selected applying a stratified two-stage cluster sampling methodology [25]. Weights adjusted for household and individual non-response among men were available in the final dataset. Details on sampling and data collection procedures have been published [25].

### Study population, analytical sample and study variables

**Fig. 1:**
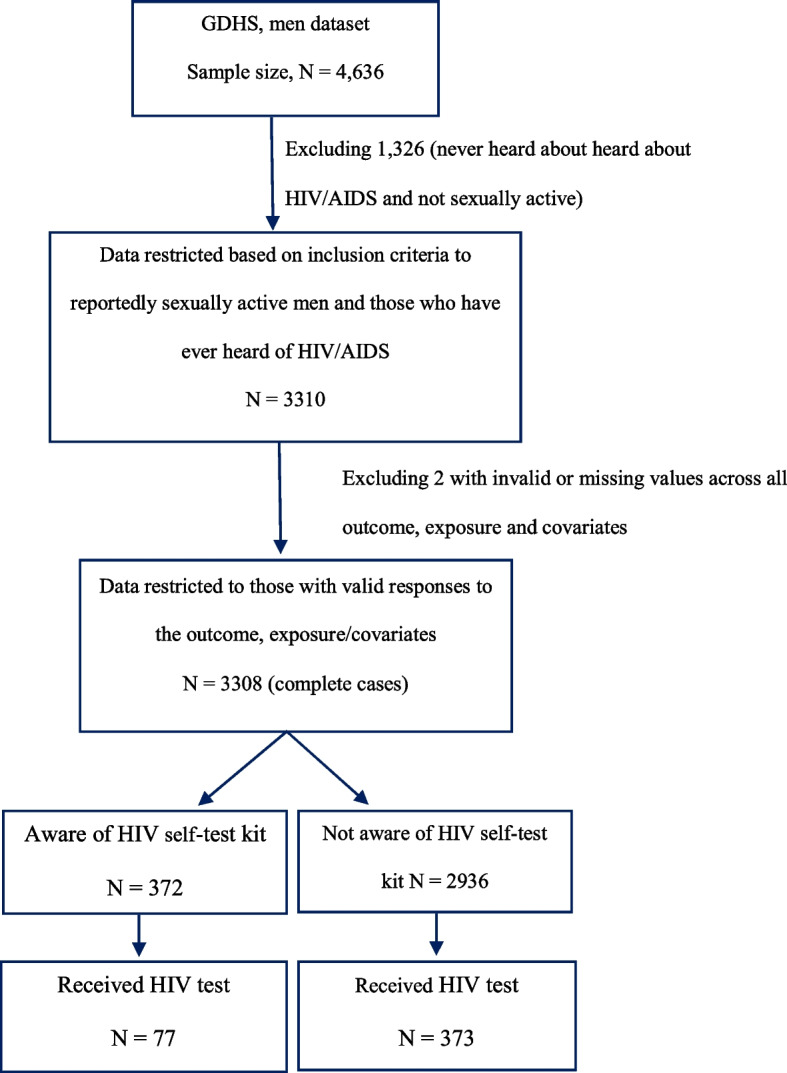
Flowchart depicting the analytical sample for association between knowledge of HIV self-test kit and recent HIV testing uptake among men using data from 2019-2020 Gambia Demographic and Health Survey

Out of 5,337 eligible men in the 2019–2020 GDHS, 4,636 completed an DHS Program’s standard men’s questionnaires, representing a response rate of 87% [25]. Of 4,636 men who participated in the survey, we included 3,310 who met our inclusion criteria: 1) participants reporting ever having been sexually active and 2) heard about HIV infection or AIDS disease. Of the 3,310 participants, only two had missing values or invalid responses such as “refused” or “don’t know” for all variables and, therefore, were excluded from the analytic sample. Information on inclusion and exclusion criteria and resulting sample sizes are shown in Fig. 1.

Recent HIV testing is the outcome variable for this analysis, defined as having had an HIV test in the last 12 months based on the survey question, “How many months ago was your most recent HIV test?” The cut-off point for 12 months was based on the reference period to monitor the global AIDS response for both the general population and people at higher risk of HIV infection [26]. Awareness of HIVST is the exposure variable for this analysis, based on a yes response to the survey question, “Have you ever heard of test kits people can use to test themselves for HIV?”.

**Fig. 2:**
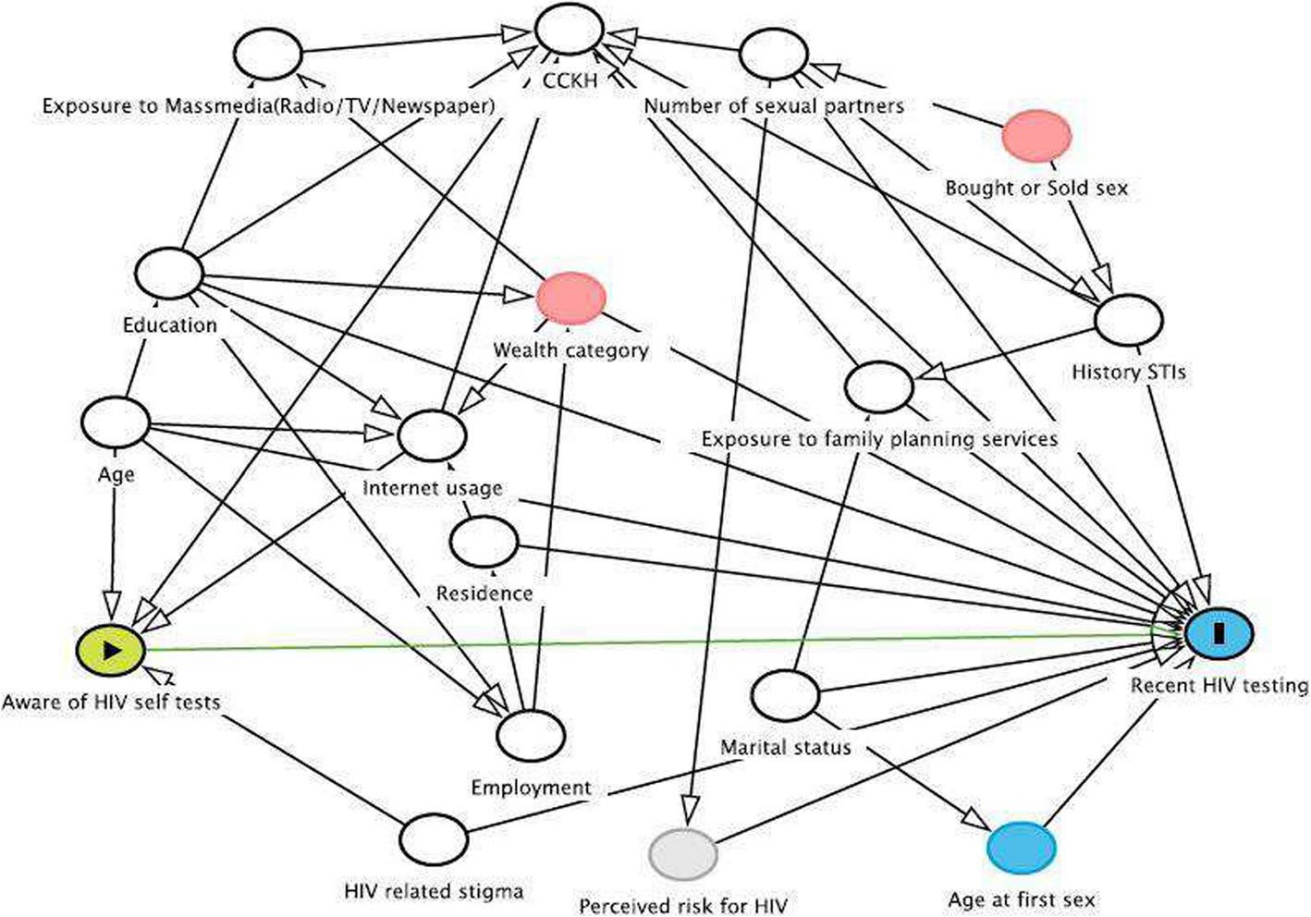
*The minimum sufficient adjustment set from DAG contains variables such as age, education, CCKH: comprehensive correct knowledge of HIV prevention methods, history of STI, HIV related stigma and internet usage

Potential confounders and risk factors of outcome variable were identified from a literature search [27–32]. In order to account for confounding pathways, we employed the best available a priori knowledge from the literature to establish the most probable causal relationship through the utilization of a directed acyclic graph (DAG) as shown in Fig. 2. A DAG is a graphical representation that illustrates relationships among variables with the exposure and the outcome, with arrows pointing in one direction (directed) and devoid of loops or cycles (acyclic) [33]. The application of a DAG allows us to identify the minimal sufficient adjustment sets, which are the key factors that need to be adjusted in order to eliminate the impact of confounding variables. By adjusting for these factors, we can more accurately assess the true cause-and-effect relationship under investigation, mitigating the potential distortion caused by confounders and confounding [34]. We have used DAGitty tool to draw this DAG to identify the minimal sufficient adjustment set [35]. Confounders included age, residence, education, comprehensive correct knowledge of HIV prevention methods (CCKH), history of sexually transmitted infections (STI) in the last 12 months, exposure to mass media (TV/Radio/Newspaper) and family planning methods, wealth index ─ detailed can be found [25], internet usage and HIV related stigma. The CCKH variable was created as a confounder according to the global definition for comprehensive knowledge of HIV prevention methods [36]. Risk factors of the outcome variable included age at first sex, marital status, employment, number of sexual partners, and whether participants bought or sold sex in the last 12 months.

### Statistical analysis

#### Primary analysis: design-adjusted logistic regression

Since the analytical dataset had < 2% of missing data, a complete cases analysis was conducted for all analyses. Survey weights, strata and clusters were used to account for the complex sampling design. Design-adjusted univariate and multivariable logistic regression (MLR) models were built [37] to estimate the association between HIVST and recent HIV testing. The variables identified in the minimum adjustment set from DAG were included into the MLR model. These variables were age, education, CCKH, history of STI, internet usage, and HIV stigma. Other variables, such as employment, marital status, residence, wealth, age at first sex, number of sexual partners, bought or sold sex in the last 12 months, exposure to mass media and family planning methods, were examined by backward elimination method to fit the final model with a minimized Akaike Information Criterion (AIC) [38]. The Archer-Lemeshow test [39] and the receiver operating characteristic curve (ROC) [40] were performed to assess the goodness of fit of the final model. All data cleaning and analyses were completed in R software version 4.0.5 [41].

#### Sensitivity analysis: propensity score weighting approach

The robustness of results from the primary analysis was evaluated with Propensity scores (PS) in weighted analysis [42]. Inverse probability weighting was used to estimate the average treatment effect (ATE) [43]. PS were estimated through logistic regression using all variables tested in the primary analysis. Survey featured variables (survey weights, strata and clusters) were added as covariates in the PS logistic model [44]. The standardized mean difference (SMD) with cut-off point of 0.2 was used to assess covariate balance [45]. Further, we employed a double-adjustment approach to minimize residual confounding by adjusting covariates included in the final MLR model of primary analysis again in outcome model [46].

## Results

### Study sample characteristics

A total of 3,308 men were included in the analysis, among which, 38% were aged between 15 to 29 years old and 78% were residents in urban areas. Most men (59%) had attained secondary or higher levels of education, reported being married or living with partners (60%,) and were in the middle or rich wealth index (68%). The vast majority of men (91%) were employed and roughly, one-third had comprehensive correct HIV knowledge. More than 15% disclosed that they had more than two sexual partners and about 4% experienced STI symptoms in the last 12 months. Only 11% of reportedly sexually active Gambian men were aware of the HIVST kit at the time of interview and 16% received HIV testing in the last 12 months (Details in Table 1).

**Table 1:** Descriptions of study population and bivariate association between recent HIV testing and potentially important variables: 2019–2020 Demographic and Health Survey data—Gambia

Variables	Overall sample *n* ^a^ (%^b^)	Did not receive HIV test in the last 12 months *n* ^a^ (%^b^)	Received HIV test in the last 12 months *n* ^a^ (%^b^)	*P*-valuea^§^
**Sample size(n)**	3308	2858	450	
**Aware of HIV self-testing kit**				
No	2936(88.9)	2563(90.2)	373(81.5)	
Yes	372(11.1)	295(9.8)	77(18.5)	< 0.001
**Age groups**				
15 – 29	1182(37.7)	1073(40.2)	109(24.0)	< 0.001
30 – 39	974(29.7)	821(29.0)	153(33.5)	
40 +	1152(32.6)	964(30.8)	188(42.5)	
**Residence**				< 0.001
Rural	1340(22.4)	1194(23.8)	146(15.0)	
Urban	1968(77.6)	1664(76.2)	304(85.0)	
**Marital Status**				< 0.001
Single	1085(37.3)	986(40.0)	99(22.4)	
Married	2153(60.6)	1814(58.1)	339(74.6)	
Previously married	70(2.1)	58(2.0)	12(3.1)	
**Education**				0.075
No education/Primary	1686(41.9)	1497(42.0)	189(36.0)	
Secondary/Higher	1622(58.9)	1361(58.0)	261(64.0)	
**Employment status**				0.001
Skilled	682(23.9)	546(22.2)	136(33.0)	
Unskilled	2416(67.2)	2129(68.9)	287(57.8)	
Other or not working	210(8.9)	183(8.9)	27(9.2)	
**Wealth group**				0.032
Poor	1521(32.3)	1352(33.7)	169(24.7)	
Middle	666(20.8)	574(20.5)	92(22.5)	
Rich	1121(46.9)	932(45.8)	189(52.8)	
**Age at first sex mean (SD)**	20.5(5.1)	20.4(5.1)	21.0(4.8)	0.055
**Number of sexual partners in the last 12 months**				
No or one partner	2733(83.5)	2387(84.8)	346(76.3)	< 0.001
More than two partners	575(16.5)	471(15.2)	104(23.7)	
**Bought or sold sex in the last 12 months**				
**No**	3232(97.5)	2791(97.5)	441(97.9)	0.652
**Yes**	76(2.5)	67(2.5)	9(2.1)	
**Had STI symptoms in the last 12 months**				
No	3152(96.2)	2720(96.2)	432(96.3)	0.911
Yes	156(3.8)	138(3.8)	18(3.7)	
**Had comprehensive correct HIV knowledge**				
No	2287(66.1)	2025(68.5)	262(52.7)	< 0.001
Yes	1021(33.9)	833(31.5)	188(47.3)	
**Exposure to family planning information in the last few months**				
No	2217(67.3)	1945(68.8)	272(59.2)	0.001
Yes	1091(32.7)	913(31.2)	178(40.8)	
**Internet access**				0.036
Almost everyday	1616(53.5)	1349(52.1)	267(61.1)	
Once a week	530(16.3)	473(16.6)	57(15.1)	
Irregular or no access	1162(30.2)	1036(31.3)	126(23.9)	
**Frequency of reading newspaper**				
At least once a week	330(12.8)	252(11.5)	78(20.0)	< 0.001
Less than once a week	474(16.7)	394(16.7)	80(17.0)	
Not at all	2504(70.5)	2212(71.9)	292(63.0)	
**Frequency of listening to radio**				
At least once a week	2320(71.1)	1987(69.8)	333(78.3)	0.006
Less than once a week	661(20.0)	588(21.3)	73(12.9)	
Not at all	327(8.9)	283(8.9)	44(8.8)	
**Frequency of watching television**				
At least once a week	2047(67.9)	1741(66.8)	306(73.4)	0.125
Less than once a week	753(21.3)	677(22.0)	76(17.4)	
Not at all	508(10.9)	440(11.2)	68(9.2)	
**Hesitance to take HIV test due to HIV related stigma**				
No or not sure	672(24.1)	598(24.6)	74(21.7)	0.496
Yes	2636(75.9)	2260(75.4)	376(78.3)	

^a^ unweighted frequency to describe the sample

^b^weighted estimates (adjusting for sampling weight, strata and sampling unit)

^§^*P*-values estimated using Thomas-Rao modification chi-square or survey-weighted t-tests, *STI* Sexually transmitted infections

### Association between recent HIV testing uptake, awareness of HIV self-kit and other variables

Men who had an HIV test in the last 12 months were older, educated, married, residing in urban areas and in a higher wealth category, more likely to have more than two partners in the last 12 months and had correct comprehensive knowledge of HIV prevention methods compared to the men who did not have an HIV test in the last 12 months. Similarly, men who had a recent HIV test were more likely to have had almost daily internet exposure and received family planning information in the last 12 months. In the bivariate analysis, recent HIV testing was significantly associated with awareness of HIVST (*p*-value < 0.001) (Details in Table 1).

### Primary analysis: design-adjusted logistic regression

In the design-adjusted univariate logistic regression analysis, men who had awareness of HIVST kits had higher odds of receiving an HIV test in the last 12 months (Odds Ratio (OR): 2.09, 95% Confidence Interval (CI): 1.52, 2.87) (Table 2). In the design-adjusted MLR, we included the variables identified in the minimum adjustment set as well as risk factors identified from the AIC backwards selection process in the final model. The final design-adjusted MLR model suggested that individuals who were aware of HIVST had 1.76 times (CI: 1.26, 2.45) the odds of undertaking an HIV test in the last 12 months compared to those who were not aware of HIVST (Table 2).

**Table 2:** Results of survey-weighted analysis assessing the relationship between awareness of HIV self-testing kit and recent HIV testing: 2019–2020 Demographic and Health Survey data—Gambia

	**Unadjusted OR**^**a**^** (95% CI)**	**Adjusted aOR**^**a**^**(95% CI)**
Primary analysis: Design-adjusted logistic regression using overall study population^b^	2.09 [1.52;2.87]	1.76 [1.26;2.45]
Design-adjusted logistic regression using data weighted with IPW ATE^c^	1.65 [1.05;2.60]	1.59 [1.03;2.45]

*OR* Odds ratio, *aOR* Adjusted odds ratio, *CI* Confidence interval. All estimates are weighted using sampling weights, sampling units and strata available in the 2019–2020 Gambia Demographic Health Survey data

^a^Adjustment variables in multivariable logistic regression models included age, residence, marital status, education, employment status, CCKH, number of sexual partners, history of STIs, internet usage, frequency of exposure to mass media, HIV stigma and exposure to family planning methods in the last few months. STI: sexually transmitted infections

^b^Survey-weighted estimates from unadjusted and adjusted logistic regression using overall study population. Adjusted model discrimination and calibration: AUC = 0.69, Archer-Lemeshow (*p* = 0.14)

^c^Weighted estimates from unadjusted and adjusted logistic regression using PS-weighted data. This data used inverse probability weights (IPW) estimating the average treatment effect (ATE). PS was estimated based on age, residence, marital status, education, CCKH, age at first sex, number of sexual partners, history of STIs, internet usage, frequency of exposure to mass media such as TV, newspaper and radio, HIV stigma, region, work category, bought or sell sex in the last 12 months, exposure to family planning methods through media in the last few months and wealth index

### Sensitivity analysis: propensity score weighted analysis

Both unadjusted and covariate-adjusted PS weighted models revealed similar estimates as in the main analysis (ATE estimates: OR: 1.65, CI: 1.05–2.60; aOR: 1.59, CI: 1.03–2.45) (Table 2).

## Discussion

### Summary of study findings

In this population-based analysis, we found that awareness of HIVST among Gambian men was low; approximately one in ten reportedly sexually active men have ever heard of HIVST. There was a significant positive association between men who were aware of HIVST and received an HIV test in the last 12 months. Specifically, Gambian men who were aware of HIVST had 76% (CI: 26%- 145%) greater odds of having had an HIV test in the last 12 months compared to those who were not aware of HIVST. Findings from both sensitivity analyses using PS weighting agreed with the main results, suggesting that HIV testing in the last 12 months differs in men who are aware of HIVST and unaware of HIVST.

### Comparison with existing literature

In Gambia, the awareness self-testing for HIV was found to be also low among reproductive women [25]. These findings may reflect Gambian’s slow progress towards developing and implementing a comprehensive HIV-ST program within the country [7]. Overall, findings from our analysis were supported by current evidence on low awareness of HIVST among men in SSA [20–23, 47]. A systematic review and meta-analysis on men’s perspectives on HIVST in SSA revealed that poor knowledge of HIVST among men did not depend on implementation of HIVST programs within the country [24, 48]. For example, the population-based trial survey data collected from 7,146 individuals in Zimbabwe found that one fifth of participants remain unaware of HIVST even after an intensive community-based door-to-door HIVST distribution campaign [48]. Nevertheless, several studies from SSA highlighted the need to promote HIVST awareness campaigns as an important intervention to increase HIVST knowledge and ensure the successful implementation within the country [22, 49].

Similarly, current evidence suggests that awareness and availability of HIVST is associated with HIV testing uptake among men in SSA, including testing with HIVST kits [50, 51]. These studies were randomized trials, particularly looking at the effect of health education on HIV testing uptake in general as well as HIV testing with self-test kits. Other cross-sectional studies also identified that men who have ever had an HIV test were more likely to be aware of HIVST [23, 52, 53]. However, a cross-sectional study conducted in Thailand found that awareness of HIVST had no association with HIV testing practice [54]. Since this finding was from an Asian general population, the results may not entirely represent men in SSA. In addition, current evidence from SSA suggests that there are many other factors, including individual risk perception, HIV-related stigma and conveniently available HIV tests, that can influence uptake of HIV testing [55]. A recent systematic review on HIVST uptake and intervention strategies revealed that HIVST is most acceptable to men in SSA [56]. Further, both quantitative and qualitative studies conducted among African men underscored the impact of HIVST awareness on high acceptability and willingness to use HIVST, particularly among the male population [20, 49, 57]. Further, HIVST acceptance was found to be consistently higher among men compared to women [58] and recent experiences from SSA countries showed high potential of HIVST promotion to reduce barriers among men and adolescents in SSA to have an HIV test, particularly for those who have never had an HIV test [4, 59]. This consistent finding highlights individual preferences for HIVST when being aware of different testing options.

### Strengths and limitations

To our knowledge, this is the first analysis in Gambia that used nationally representative male individuals’ data to examine awareness of HIVST and whether prior HIVST awareness increases HIV testing uptake in the last 12 months. Findings from our analysis are relevant, considering that the national HIV control program recently launched “the Gambia Catch-up plan” for more intense HIV case identification and high-quality HIV antiretroviral therapy [7]. Since we employed data from a nationally representative study, our findings may be considered to be generalizable to the 2019–2020 Gambian male population.

In addition, our findings can be considered to be robust as observed associations were statistically significant and consistent in sensitivity analyses using the PS weighting method. To mitigate the residual confounding, we adopted a double-adjustment approach after weighting based on PS, by adjusting for covariates again in the design-adjusted MLR outcome models. Applying the PS-weighting approach, conditional ATE was estimated for potential outcomes over the entire Gambian male population. Findings from this PS-weighted analysis will be particularly useful for nationwide HIVST program planning and implementation.

This study has several limitations. Since data were collected from a cross-sectional study, temporality is difficult to establish [60]. With existing variables in the database, the issue around temporarily could not be addressed. However, since the HIVST program in Gambia is still in its infancy, a significant association from our analysis will be essential to facilitate the development of strategies for HIVST program implementation, regardless of the direction of association. Second, the use of secondary data from GDHS 2019–2020 may not adequately control for important confounders due to the availability of existing variables in the dataset. For example, health-seeking behavior and perceived HIV risk are known to be associated with both HIVST awareness and HIV testing uptake [30, 61] but were unmeasured. Since these confounders could not be included in the analysis, we accounted for such unmeasured confounding using exposure to family planning services as proxy variables. Third, our GDHS data were based on self-reported measures; therefore, the responses are prone to recall bias and social desirability bias, both of which are more likely to lead to an underestimation of true association of interest [62]. However, our exposure and outcome were derived from questions assessing current HIVST awareness and recent testing; therefore, it is less susceptible to reporting bias associated with recall. Fourth, we have limited availability to explore source of HIVST information, as the original survey did not have follow-up questions regarding the awareness of HIVST. Finally, detailed information related to HIVST initiatives piloted in Gambia, including specific types of HIV test kits used was not available, therefore, we were unable to comment further on types of available tests and HIVST related instruction on testing practices.

### Policy implication and future research direction

Our findings are significant as well as timely because it highlights the potential effects of prioritizing HIVST awareness programs on the uptake of HIV testing, especially in the stage of incorporating an HIVST strategy into the national HIV control program. Increased knowledge of HIV status through HIVST is essential for Gambian men, along with their partners, to receive needed care and treatment, initiate safer sex and to reduce HIV transmission in the community. Successful programs to increase HIVST awareness among men include offering HIVST at formal and informal workplaces [63], through peer outreach, and through internet recruitment and the distribution of tests by mail [64, 65]. However, we were not able to explore the availability of HIVST, its usage, and linkage of self-testing results to the HIV treatment and care cascade. Based on our preliminary findings, further studies are warranted to explore preferences for HIVST utilization and the linkage to HIV care and communication strategies with clear instructions on HIVST use for the successful implementation of an HIVST program in Gambia.

## Conclusion

We found that awareness of HIVST among reportedly sexually active men was low; those with awareness of HIVST were more likely to have had an HIV test in the last 12 months, and this association was statistically significant. Given the country’s current adoption of an HIVST program, our findings will help prioritize planning and awareness-raising activities towards men, particularly men who have never had an HIV test. These findings accentuate the need to promote awareness of HIVST as an important intervention for increasing the uptake of HIV testing among Gambian men who traditionally have had low uptake of HIV testing.

## Data Availability

Data is publicly available from Demographic and health survey at https://dhsprogram.com/
